# A semisynthetic, multicofactor artificial metalloenzyme retains independent site activity

**DOI:** 10.1007/s00775-025-02095-z

**Published:** 2025-02-01

**Authors:** Ashlee E. Wertz, Ilmari Rosenkampff, Philippe Ibouanga, Matthias Huber, Corinna R. Hess, Olaf Rüdiger, Hannah S. Shafaat

**Affiliations:** 1 Department of Chemistry and Biochemistry, The Ohio State University, 100 W 18th Ave, Columbus, OH 43210 USA; 2 Max Planck Institute for Chemical Energy Conversion, Stiftstrasse 34-36, 45470 Mülheim an der Ruhr, Germany; 3 Department of Chemistry and Catalysis Research Center, Technical University of Munich, 85748 Garching, Germany; 4 Institut de Chimie Moléculaire et des Matériaux d’Orsay, Université Paris-Saclay, CNRS, 91405 Orsay, France; 5 Faculty of Chemistry and Pharmacy, University of Regensburg, 93053 Regensburg, Germany; 6 Department of Chemistry and Biochemistry, University of California, Los Angeles 607 Charles E. Young Drive East, Los Angeles, CA 90095 USA

**Keywords:** Hydrogenase, Redox cofactor, Electron transfer, Bioorthogonal coupling, Electrocatalysis

## Abstract

Native metalloenzymes are unparalleled in their ability to perform efficient small molecule activation reactions, converting simple substrates into complex products. Most of these natural systems possess multiple metallocofactors to facilitate electron transfer or cascade catalysis. While the field of artificial metalloenzymes is growing at a rapid rate, examples of artificial enzymes that leverage two distinct cofactors remain scarce. In this work, we describe a new class of artificial enzymes containing two different metallocofactors, incorporated through bioorthogonal strategies. Nickel-substituted rubredoxin (Ni^Rd^), which is a structural and functional mimic of [NiFe] hydrogenases, is used as a scaffold. Incorporation of a synthetic bimetallic inorganic complex based on a macrocyclic biquinazoline ligand (M^MBQ^) was accomplished using a novel chelating thioether linker. Neither the structure of the Ni^Rd^ active site nor the M^MBQ^ were altered upon attachment, and each site retained independent redox activity. Electrocatalysis was observed from each site, with the switchability of the system demonstrated through the use of catalytically inert metal centers. This M^MBQ^–Ni^Rd^ platform offers a new avenue to create multicofactor artificial metalloenzymes in a robust system that can be easily tuned both through modifications to the protein scaffold and the synthetic moiety, with applications for redox catalysis and tandem reactivity.

**Graphical abstract:**

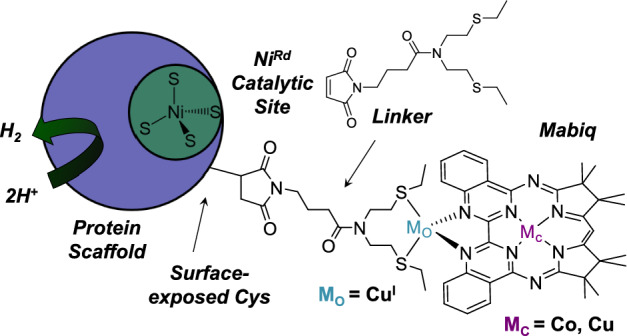

**Supplementary Information:**

The online version contains supplementary material available at 10.1007/s00775-025-02095-z.

## Introduction

For decades, chemists have endeavored to develop viable catalysts for small molecule activation reactions—e.g., H_2_ evolution, CO_2_ conversion, and N_2_ fixation [1, 2] all of which can effectively be catalyzed by metalloenzymes, e.g., hydrogenases [3], carbon monoxide dehydrogenases [4], and nitrogenases [5]. The remarkable activity of the enzymes stems not only from the metal type and first coordination sphere but from the protein environment far beyond [6]. Second coordination sphere components, such as H-bonds and charged functionalities, likewise can enhance the catalytic performance of molecular catalysts [7, 8]. However, the beneficial features conferred by the protein scaffold are challenging to fully incorporate in synthetic compounds. In this regard, artificial enzymes are a particularly attractive class of small molecule activation catalysts [9]. Artificial enzyme constructs are generally based on small, robust proteins that allow for facile tuning of the wider coordination sphere environment [10–13].

**Fig. 1:**
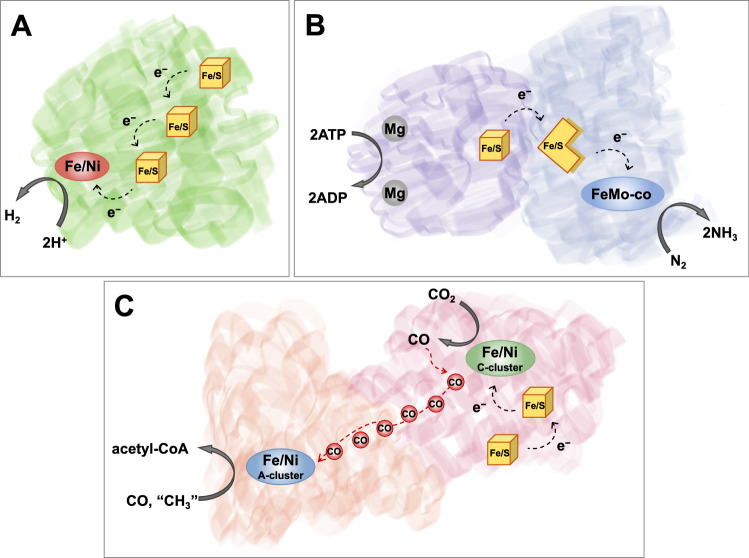
Native metalloenzymes for small molecule chemistry rely on multiple metal cofactors for catalysis. Small molecule binding and activation occurs at a multimetallic site. The **A** hydrogenases, **B** nitrogenases, and **C** CODH additionally use Fe/S clusters for electron transfer. N_2_ activation by the nitrogenases is also dependent on Mg-catalyzed ATP hydrolysis. CO generated by CODH is used for CoA acetylation in a tandem reaction with acetyl CoA synthase

A challenge is that the native metalloenzymes frequently rely on the cooperative function of multiple metal cofactors [14]. These include combinations of substrate activation and/or electron transfer sites, which are often several ångstroms apart (Fig. 1). For example, while H_2_ and protons bind to binuclear metal centers of the [FeFe] and [NiFe] hydrogenases, a series of iron–sulfur clusters is required to shuttle electrons between soluble redox partners and the catalytic active site [15–17]. The conversion of N_2_ to NH_3_ at the FeMoco site of nitrogenase is coupled to Mg-catalyzed ATP hydrolysis, which occurs in the Fe–protein component over 30 Å away [18]. The necessary electrons for N_2_ reduction are again transported via Fe/S clusters. Carbon monoxide dehydrogenases (CODHs) use Fe/S clusters and an Fe/Ni/S cluster for electron transfer and CO_2_ activation, respectively [4]. Furthermore, in bifunctional Ni–CODHs, the CO generated at the CODH active site is subsequently used for the carbonylation of CoA by yet another Fe/Ni site of the acetyl CoA synthase (ACS) component. The latter provides a particularly intriguing example of tandem catalysis.

Despite the advantages of artificial metalloenzymes and impressive advancements in their design [9, 10, 19], constructs containing multiple metal active sites are rare—particularly constructs containing several catalytic centers [20–23]. Yet, the development of artificial enzymes with multiple cofactors could be a step change for catalysis. As is apparent from studies on hydrogenases, the incorporation of well-matched electron transfer sites is crucial for catalytic efficiency [24]. The presence of multiple metal cofactors at well-defined sites that can activate different substrates would also offer new opportunities for protein-based tandem catalysis.

**Fig. 2:**
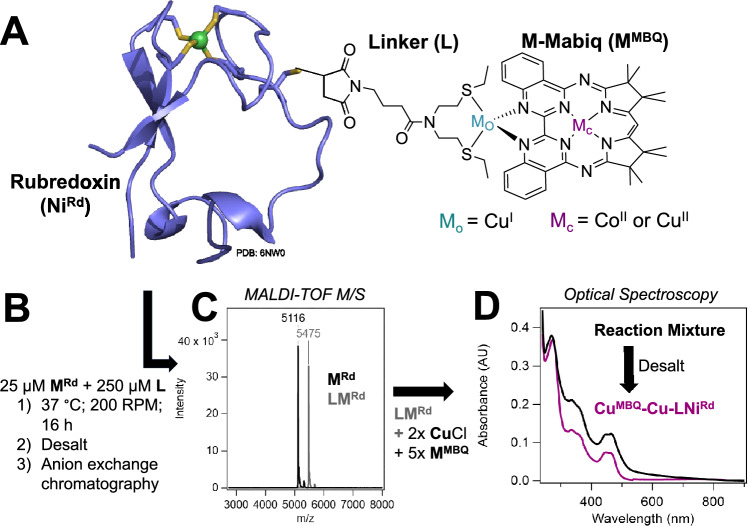
**A** Cartoon depiction of M^MBQ^–Cu–LNi^Rd^ construct. **B** Reaction scheme for formation of LM^Rd^ and M^MBQ^–Cu–LM^Rd^ constructs showing **C** MALDI-TOF mass spectrometry analysis for verification and **D** representative optical spectra of the reaction mixture and purified Cu^MBQ^–Cu–LNi^Rd^

We herein describe a new rubredoxin-based artificial enzyme construct, M^MBQ^–Cu–LM^Rd^ (Fig. 2), containing two distinct metal active sites, incorporated via orthogonal strategies. The first site, M^Rd^, consists of a Ni or Zn center coordinated by four cysteines, generated via substitution of the native Fe center of rubredoxin (Rd). Ni^Rd^ is a functional model of [NiFe] hydrogenases that is capable of both electrochemical and solution-phase hydrogen evolution [25, 26]. Additional benefits of this artificial enzyme include a scaffold that retains fold and activity even upon extensive modification [27], the ability to attach synthetic components to a native cysteine residue near the active site [28, 29], and stability in a variety of solvents and solvent mixtures.

The second metal site of our construct is comprised of a synthetic bimetallic inorganic complex based on a macrocyclic biquinazoline ligand, M^MBQ^ (Fig. 2). M^MBQ^ contains two metal binding sites, one in the macrocyclic cavity (M_c_) and one in the outer biquinazoline unit (M_o_). The complex was attached to M^Rd^ via a novel strategy using a chelating thioether linker attached to both M_o_ and a surface Cys residue of the protein. The coordination chemistry and redox properties of M^MBQ^ have been extensively studied in solution, with the one-electron reduction potential of Co^MBQ^ aligning well with that of Ni^Rd^ [30–33]. A range of metals can be incorporated into the central binding site (M_c_) of M^MBQ^, allowing for various reactivities, fine tuning of the redox properties, as well as distinct photochemical properties [34]. The peripheral metal binding site (M_o_) provides increased modularity. Previous studies have also shown that the Co^MBQ^ complex acts as an electrocatalyst for H_2_ evolution [35], while Fe^MBQ^ and the binuclear Fe^MBQ^Cu complexes can electrocatalytically and photocatalytically reduce CO_2_ [36]. The demonstrated versatility of the M^MBQ^ complexes along with the opportunity for varying the modality of attachment to the protein suggested potential for synergistic electronic communication between the redox sites.

For the purposes of the current study, M^MBQ^ complexes were examined with Cu^I^ at M_o_, and either Co^II^ or Cu^II^ at M_c_. Optical and X-ray absorption spectroscopies (XAS) indicate that the electronic and geometric structures of the metal centers are not disrupted upon incorporation of M^MBQ^ onto M^Rd^. Importantly, the Ni^Rd^ and Co^MBQ^ sites both retain H_2_ evolution activity. The activity of each site can be switched off using Zn^Rd^ or Cu^MBQ^, respectively. This study establishes the M^MBQ^–Cu–LM^Rd^ construct as an artificial enzyme assembly with distinctive functional active sites. The system allows for the development of bifunctional catalysts as well as studies on the effect of electron relays on catalysis.

## Materials and methods

### Linker synthesis

The synthetic scheme for the thioether linker is shown in Scheme S1. Reagents 4-(2,5-dioxo-2,5-dihydro-1H-pyrrol-1-yl)butanoic acid and bis(2-(ethylthio)ethyl)amine hydrochloride were synthesized according to literature procedures [37, 38].

#### 4-(2,5-Dioxo-2,5-dihydro-1H-pyrrol-1-yl)-N,N-bis(2-(ethylthio)ethyl)butanamide (Linker, L)

1 g (5.46 mmol) 4-(2,5-Dioxo-2,5-dihydro-1H-pyrrol-1-yl)butanoic acid was dissolved in 50 mL DCM in a Schlenk flask equipped with a magnetic stir bar and placed under argon. The reaction mixture was cooled to 0 °C and 4.1 ml (54.6 mmol) SOCl_2_ were subsequently added dropwise to the solution over 30 min. After 3 h, the volatiles were removed under vacuum, leaving a yellow powder that was redissolved in 50 mL DCM. A solution of bis(2-(ethylthio)ethyl)amine hydrochloride (4.96 mmol) and NEt_3_ (8.19 mmol) in 30 mL DCM was added dropwise to the solution and the mixture was stirred overnight. The reaction mixture was washed with 3 × 50 mL 1 M NaHCO_3_, followed by 3 × 1 M HCl. The aqueous phases were extracted three times with EtOAc, and the combined organic phases were washed with brine and dried over MgSO_4._ The volatiles were removed in vacuo to yield a brown oil. The product was purified by column chromatography on two consecutive silica columns (1: DCM with a gradient 0% MeOH to 4%, R_f _= 0 to 0.8; 2: Hexane:EtOAc 3:2, Rf = 0.35) yielding L as an off-white oil (750 mg, 42%). L was characterized by ^1^H NMR, ^13^C NMR and ESI–MS (Figs. S1–3) confirming the integrity of the compound. ^1^H NMR (400 MHz, CDCl_3_) δ 6.69 (s, 2H), 3.60 (t, *J* = 6.6 Hz, 2H), 3.47 (dt, *J* = 8.1 Hz, 4H), 2.69–2.61 (m, 4H), 2.57 (dt, *J* = 7.3 Hz, 4H), 2.35 (t, *J* = 7.2 Hz, 2H), 1.95 (p, *J* = 6.9 Hz, 2H), 1.27 (dt, *J* = 7.5, 2.7 Hz, 6H); ^13^C NMR (101 MHz, CDCl_3_) δ 171.37, 170.92, 134.19, 48.71, 46.76, 37.51, 30.35, 29.23, 26.52, 26.13, 24.14, 15.00, 14.97. ESI–MS [M + H]^+^ (*m/z*: 359.144228, calc. 359.145761), [M + Na]^+^: (*m/z*: 381.126273, calc. 381.127705).

### Covalent attachment of linker to rubredoxin (LM^Rd^)

M^Rd^ was expressed, purified, and metalated as previously described [25, 26]. Possible intermolecular disulfide bonds at C31 were reduced by the addition of dithiothreitol (DTT) to a final concentration of 2.4 x [M^Rd^], followed by incubation at room temperature for 5–10 min. After incubation, excess DTT was removed via an Econo-Pac 10-DG desalting column. The labeling reactions were then prepared by dilution of M^Rd^ to a final concentration of 25 µM M^Rd^ and addition of L to a final concentration of 250 µM. Reaction mixtures were incubated at 37 °C, with shaking at 200 RPM, for 16 h in a 1:1 mixture of 50 mM Tris buffer, pH 8.0, and dimethylformamide (DMF). Protein stability in DMF was verified through optical spectroscopy. After incubation, the labeling reactions were concentrated by a 3 kDa MWCO Amicon stirred cell (Millipore Sigma, St. Louis, MO) and Centricon ultrafiltration devices to approximately 500 µL. Excess linker was removed using a desalting column. A HiTrap Q Sepharose anion exchange column was used to remove unlabeled protein from the reaction mixture, and successful generation of the linked complex was confirmed via MALDI-TOF mass spectrometry (Fig. 2). No change in the optical absorption features of Ni^Rd^ was seen with the linker attachment (Fig. S4). Identical protocols were followed for Ni^Rd^ and Zn^Rd^.

### M^MBQ^–Cu–LM^Rd^ complex formation

All manipulations were carried out under anoxic conditions within an anaerobic chamber (MBraun). The M^MBQ^–Cu–LM^Rd^ constructs were formed by incubating 1 mM LM^Rd^ with 2 mM CuCl (Thermo Fisher) and 5 mM M^MBQ^ for 10 min, in 1:1 DMF:50 mM Tris pH 8.0 buffer then removing excess M^MBQ^ and CuCl with an Econo-Pac 10-DG desalting column. The complexes were centrifuged at 5000*xg* for 10 min to remove any unreacted M^MBQ^ that precipitated out of solution, and the desired supernatant was pipetted off. Labeling efficiency and concentration were determined by UV–Vis spectroscopy. The concentration of Ni^Rd^ was determined using the known extinction coefficient of the d–d absorption bands for the Cu^MBQ^–Cu–LNi^Rd^ construct. A bicinchoninic acid (BCA) protein assay kit (Sigma-Aldrich) was used to determine concentration of the Co^MBQ^–Cu–LNi^Rd^ construct as the absorption bands originating from Ni^Rd^ were masked by strong absorption features from the Co^MBQ^ (Fig. S5).

### X-ray absorption spectroscopy

For X-ray spectroscopy, solutions containing 1.0 mM Cu^MBQ^–Cu–LZn^Rd^, 390 µM Co^MBQ^–Cu–LNi^Rd^, 830 µM Co^MBQ^–Cu–LNi^Rd^, 720 µM Co^MBQ^–Cu–LZn^Rd^ and 470 µM Cu^MBQ^–Cu–LNi^Rd^ solutions were prepared in 50 mM Tris buffer, pH 8.0, with 30% glycerol and frozen in liquid nitrogen for measurements. These concentrations were measured from optical spectroscopy and represent multiple sample preparations. EXAFS and XAS K-edges of the MBQ-containing samples were measured at the SAMBA beamline of the SOLEIL synchrotron in France with a sagittally bent double crystal Si(220) monochromator and a 33-element germanium detector with an approximate photon flux of 1 × 10^11^ ph/s. A Cu filter was used for Zn K-edge measurements except for measurements of Co^MBQ^–Cu–LZn^Rd^, and a Co filter was used for Ni K-edge measurements to minimize background from the elastic peak. The Ni^Rd^ sample was measured at the P64 beamline of the Positron–Electron Tandem Ring Accelerator (PETRA III) at the Deutsches Elektronen-Synchrotron (DESY) (100 mA, 5 GeV). Ni K-edge XAS spectra were recorded at an electron energy of 8.2–8.9 keV, using a fluorescence single-element silicon drift Vortex detector. The spectra were calibrated to the maximum of the lowest energy peak in the first derivative spectra of the metal foil in transmission. The reference values used were 7709 eV for Co, 8333 eV for Ni, and 9659 eV for Zn. Spectra were measured in partial fluorescence yield mode at the respective Kα lines. Data were analyzed using FEFF software and the IFEFFIT package using the Artemis software included in the Demeter package [39].

### MALDI-TOF mass spectrometry

MALDI-TOF samples were prepared on a ground steel plate (Bruker MSP 96 microScout Target) using a matrix made of 100 mM sinapinic acid (Sigma) in 70%/30% v/v water/acetonitrile with 0.1% trifluoroacetic acid. For all samples, 1 µL of sample/matrix was deposited onto the plate and allowed to dry completely before analysis. All measurements were carried out with a Bruker microFlex MALDI-TOF mass spectrometer operating in linear-positive mode.

### Electrochemistry

All electrochemistry experiments were carried out in a nitrogen glovebox. For control experiments involving only a bimetallic Mabiq (LCu–M_c_^MBQ^), a graphitic electrode was first modified with in situ*-*generated aryl diazonium salt of 4-aminothiophenol as described previously [40]. This electrode modification was selected to have a comparable M^MBQ^ attachment modality to the electrode as when the M^MBQ^ is coupled with the M^Rd^. Excess linker was removed by rinsing with DMF. 7 µl of 4 mM [Cu(MeCN)_4_][PF_6_] in DMF was pipetted on the electrode surface, and after 2 min, an equal amount of 10 mM M^MBQ^ dissolved in DMF was introduced and the mixture left to react for 10 min. To validate the covalent attachment, XPS of LCu–Co^MBQ^ covalently attached on a glassy carbon plate was measured, showing transitions from Cu, Co, and S (Fig. S6). Three types of electrodes were measured: electrode LCu, containing the linker and Cu^I^ from [Cu(MeCN)_4_][PF_6_], electrode L–M^MBQ^, containing the linker and dropcasted M^MBQ^, and electrode LCu–M^MBQ^, containing the linker, Cu^I^, as well as M^MBQ^ (Fig. S7). Prior to electrochemical measurements, the electrode was rinsed with DMF. A saturated calomel electrode (SCE) was used as the reference electrode, and a platinum wire was used as the counter electrode. Potentials were reported relative to the normal hydrogen electrode (NHE) by addition of 0.24 V.

For electrochemistry on the M^MBQ^–Cu–LM^Rd^ samples, pyrolytic graphite (PG) working electrodes (thegraphitestore.com) were polished with sandpaper and sonicated for 180 s in distilled water. Samples were electrostatically adsorbed to the electrode by aliquoting 10 µL of 50 µM M^MBQ^–Cu–LM^Rd^ construct onto the PG electrode, allowing the solution to sit for 30 s and then removing any excess liquid. All electrochemistry experiments were run in CHaMp buffer (25 mM each **C**HES, **H**EPES, sodium **a**cetate, and **M**ES, with 50 mM sodium **p**erchlorate) at the desired pH value. A Ag/AgCl reference electrode was used with a platinum wire counter electrode; potentials were converted to NHE by addition of 0.198 V. The onset potential was obtained as described previously, using the inflection point of the first derivative of the voltammogram [26]. The anodic and cathodic M^MBQ^ peak positions were determined using the qSOAS program [41]. Error bars represent ± 1 standard deviation across n = 3 trials.

TOF values were obtained as outlined in Ref. [42]. This protocol follows the evolution of the current density of the protein adsorption, which displays an initial linear time dependence that is proportional to the TOF, the concentration of the enzyme in solution, and the diffusion coefficient of the enzyme. For more information on the relevant parameters, see the Supplemental Information (Appendix 1). In each of the experiments, a rotation rate of 1000 rpm was used with freshly polished PG rotating disk electrodes (RDEs). For determining current density, the geometric surface area of the PG electrode was used. Experiments were carried out at 25 ˚C, in pH 3 CHaMp buffer, with a solution protein concentration of 0.3 µM. To remove catalytically active metal impurities from the buffer, the buffer was stirred with Chelex-100 resin (5 g/100 ml) overnight. Subsequently, the mixture was filtered through a paper filter, and the pH was adjusted.

### Absorption spectroscopy

UV–Visible optical spectra were measured using a Shimadzu UV-2600 spectrophotometer.

## Results

### Generation and spectroscopic characterization of M^MBQ^–Cu–LM^Rd^ constructs

**Fig. 3:**
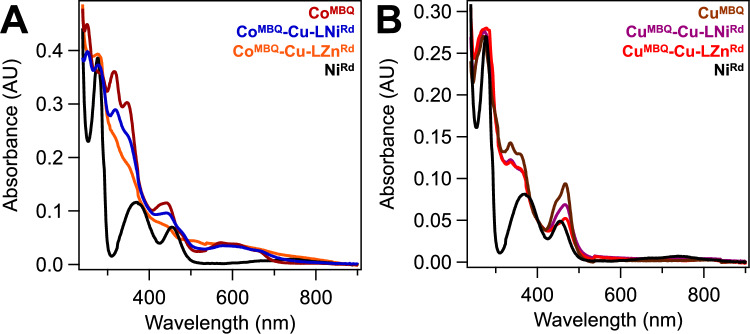
Optical spectra of M^MBQ^–Cu–LM^Rd^ constructs for (**A**) Co^MBQ^ constructs and (**B**) Cu^MBQ^ constructs. All solutions were measured in 50 mM Tris buffer, pH 8.0, except for Co^MBQ^, which is shown in DMF. M^MBQ^ and Ni^Rd^ are shown for comparison

Co^MBQ^–Cu–LM^Rd^ and Cu^MBQ^–Cu–LM^Rd^ constructs were formed with the thioether linker (**L**) covalently attached to the protein at the C31 residue and Cu^I^ as the outer metal (M_o_) in the M^MBQ^ compound. The outer metal serves to ligate the fragments but is not redox-active, as previously shown [32]. Both Ni^Rd^ and Zn^Rd^ constructs were made, as Zn^Rd^ serves as a redox-inert but structurally homologous control for the catalytically active Ni^Rd^ [43]. Labeling efficiency of the M^MBQ^ to the M^Rd^ was determined to be nearly quantitative for the Co^MBQ^–Cu–LNi^Rd^ and Cu^MBQ^–Cu–LZn^Rd^ constructs using optical spectroscopy (Fig. 3, Fig. S5) and ICP-MS (Table S1). Less than stoichiometric amounts of M^MBQ^ were present in the Co^MBQ^–Cu–LZn^Rd^ and Cu^MBQ^–Cu–LNi^Rd^ samples, with 60% and 35% labeling, respectively. No M^MBQ^–Cu–LM^Rd^ construct was formed with either Cu^MBQ^ or Co^MBQ^ in the absence of Cu^I^ (Fig. S8). Minimal amounts of M^MBQ^–Cu–M^Rd^ construct were observed when the thioether linker was not present, which was attributed to direct interactions between the bimetallic MBQ and the C31 thiolate. No labeling was observed when this cysteine residue was mutated to an alanine (C31A Rd; Fig. S8).

**Fig. 4:**
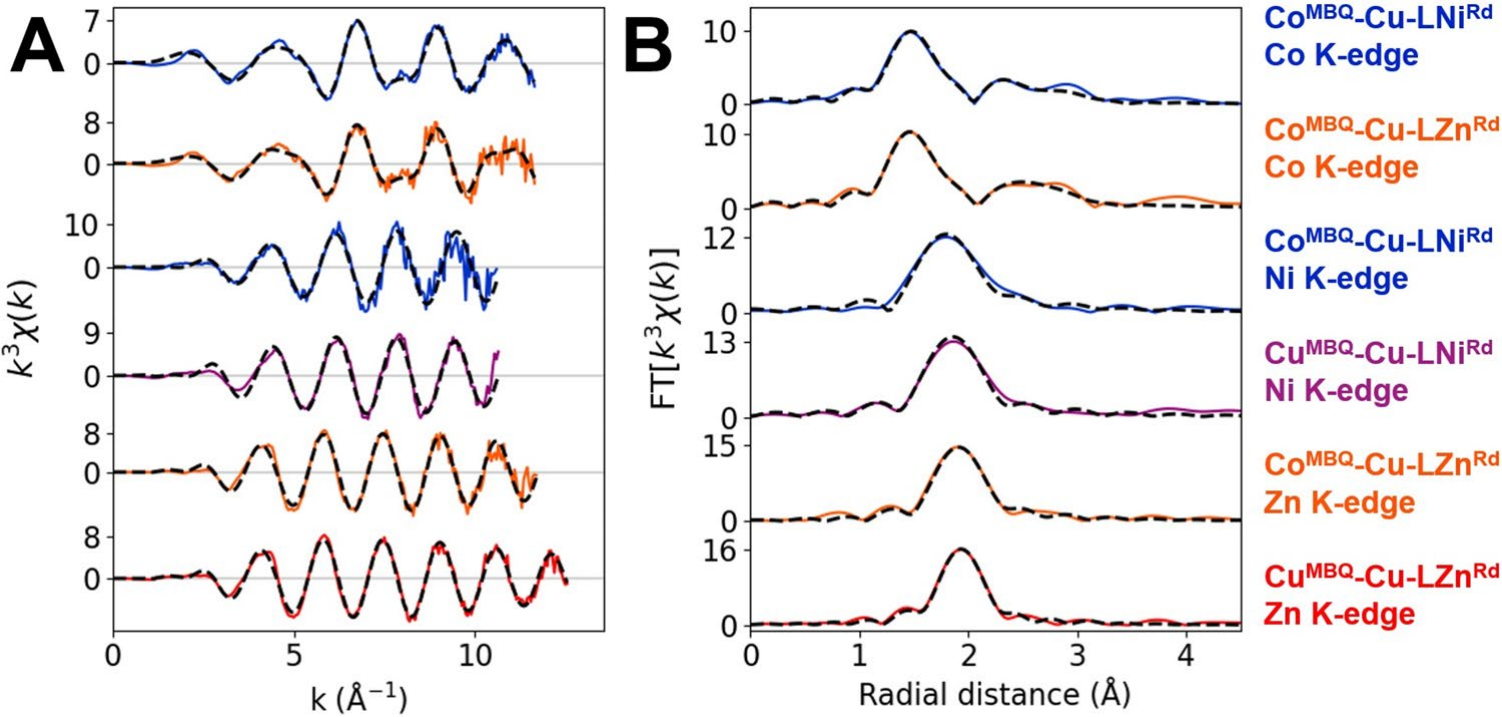
**A**) k^3^ weighted EXAFS spectra and (**B**) Fourier transforms of Co, Ni, and Zn K-edges of each M^MBQ^–Cu–LM^Rd^ construct (*solid colored traces, as indicated*). Best fits to the data are overlaid (*dashed grey traces*)

EXAFS traces of the Co, Ni, and Zn K-edges of the M^MBQ^–Cu–LM^Rd^ constructs were accurately fitted with models including the primary coordination sphere of the rubredoxin and atoms up to the third coordination sphere of Co^MBQ^ (Fig. 4). The fitted radial distances to the scatterers match within experimental error to the reported radial distances of the corresponding crystal structures [27, 44, 45], implying that the metal coordination remains intact in M^Rd^ and M^MBQ^ upon construct formation (Table S2). Furthermore, the XANES spectrum of Cu^MBQ^–Cu–LNi^Rd^ is very similar to that of the isolated Ni^Rd^, with a well-resolved intense pre-edge resulting from the tetrahedral coordination of the Ni at the active site (Fig. S9).

### Co^MBQ^–Cu–LM^Rd^ constructs are active for electrochemical proton reduction

**Fig. 5:**
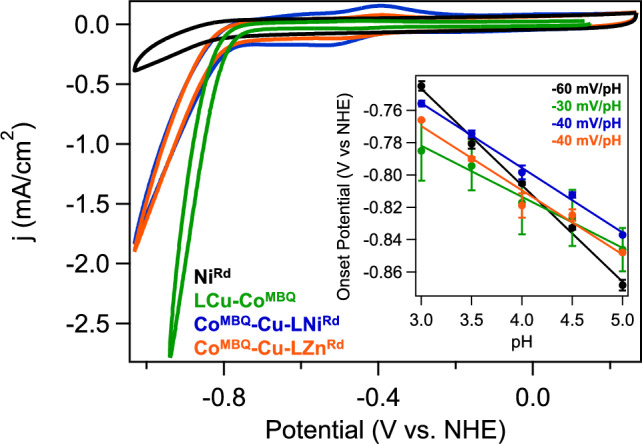
CVs of Ni^Rd^ and the Co^MBQ^ constructs at pH 4.5 in 25 mM CHAMP buffer ($\nu$  = 100 mV/s). (Inset) pH-dependent onset potentials for all systems (colored as in main panel). Lines shown reflect the indicated slopes

The electrochemistry of the covalently attached LCu–Co^MBQ^ compared to L–Co^MBQ^ shows that the presence of Cu^I^ in the linker shifts the formal Co^II/I^ redox potential of Co^MBQ^ to slightly more negative values (Fig. S10). Both LCu–Co^MBQ^ and L–Co^MBQ^ are active for aqueous electrochemical proton reduction without the Ni^Rd^ catalyst present. The Co^MBQ^–Cu–LNi^Rd^ and Co^MBQ^–Cu–LZn^Rd^ complexes also show proton reduction activity on an electrode surface (Fig. 5), with similar catalytic currents observed from both constructs. As Zn^Rd^ is redox-inert, the observed catalysis is suggested to originate from the Co^MBQ^ moiety. Further evidence that the catalysis originates predominately from Co^MBQ^ is found in (i) the similarity in waveshape between the Co^MBQ^–Cu–LZn^Rd^ and Co^MBQ^–Cu–LNi^Rd^ constructs (Fig. S11), (ii) the pH dependence of the onset potentials (Fig. 5, *inset*), and (iii) continued catalytic activity above pH 6, where Ni^Rd^ is inactive (Fig. S12–S14) [46]. Interestingly, the onset potential of catalysis appears to be affected by the M^Rd^ active-site metal, with a slightly lower overpotential observed for the Co^MBQ^–Cu–LNi^Rd^ construct than the Co^MBQ^–Cu–LZn^Rd^ construct in the pH range where Ni^Rd^ is active **(**Fig. 5**)**. We note that the Ni^Rd^ protein has been shown to be stable under all the experimental conditions probed [25, 26, 47].

Turnover frequency (TOF) analyses of activity were carried out starting from homogenous samples using a method developed by Fourmond and coworkers to study hydrogenase enzymes [42]. This method provides a useful direct comparison between the activities of the constructs. The turnover frequencies were evaluated at pH 3 and at -0.88 V vs. NHE, that is, at a more negative potential than the catalytic H^+^ reduction onset for both Ni^Rd^ and Co^MBQ^. As expected for the combination of two active hydrogen-evolving catalysts, Co^MBQ^–Cu–LNi^Rd^ shows the highest activity (15.2 ± 0.9 s^−1^); moreover, this activity is close to the sum of the individual activities of Ni^Rd^ (5.9 ± 0.1 s^−1^) and Co^MBQ^–Cu–LZn^Rd^ (8.9 ± 0.9 s^−1^) (Figs. S15–16). However, the onset potential of Co^MBQ^–Cu–LNi^Rd^ at pH 3 is similar to that of Ni^Rd^, whereas at pH 5, it is closer to that of the Co^MBQ^ moiety. This would be consistent with Ni^Rd^ and Co^MBQ^ governing the catalytic onset of Co^MBQ^–Cu–LNi^Rd^ at pH 3 and 5, respectively. Collectively, these observations indicate that both sites remain independent and active in the construct, validating the intent to generate a multicofactor artificial metalloenzyme.

### Ni^Rd^-based catalysis is observed with the Cu^MBQ^–Cu–LNi^Rd^ construct

**Fig. 6:**
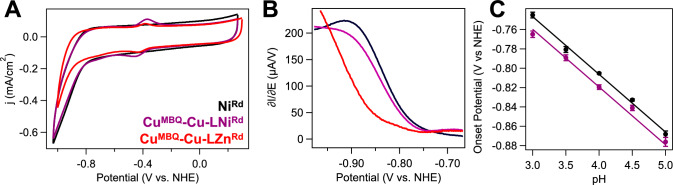
**A** CVs and **B** first derivatives of the CVs of Ni^Rd^ (black), Cu^MBQ^–Cu–LNi^Rd^ (purple), and Cu^MBQ^–Cu–LZn^Rd^ (red) constructs at pH 4.5 in 25 mM CHAMP buffer ($\nu$  = 100 mV/s). (**C**) Onset potentials as a function of pH for Cu^MBQ^–Cu–LNi^Rd^ (purple) and Ni^Rd^ (black). Lines drawn represent a – 59 mV/pH unit slope

The Cu^MBQ^–Cu–LNi^Rd^ construct was also generated to directly monitor the impact of the M^MBQ^ on Ni^Rd^ catalysis, as Cu^MBQ^ is inactive towards H_2_ evolution in the relevant potential range. In the absence of M^Rd^, the formal Cu(II/I)^MBQ^ couple is shifted to more negative values when M_o_ = Cu^I^ (Fig. S17), but the system remains catalytically inactive. On the other hand, the Cu^MBQ^–Cu–LNi^Rd^ construct is active for proton reduction, with catalytic waveshapes that closely match those of Ni^Rd^ (Fig. 6A). In the Cu^MBQ^–Cu–LZn^Rd^ construct, little signal above baseline is observed, and the characteristic sigmoidal derivative waveshape is absent (Fig. 6B). These observations are consistent with catalysis deriving from Ni^Rd^ rather than the Cu^MBQ^ moiety. Small shifts in the onset potential are observed for the Cu^MBQ^–Cu–LNi^Rd^ construct relative to Ni^Rd^ (Fig. 6C, Figs. S18–S19), while the formal Cu(II/I)^MBQ^ couple shifts anodically upon attachment to the M^Rd^ scaffold.

## Discussion

### Each site in M^MBQ^–Cu–M^Rd^ retains independent functionality and shows limited electronic communication

The redox-active Cu^MBQ^ and catalytically active Co^MBQ^ have successfully been attached to the artificial enzyme, Ni^Rd^, as a strategy to develop multicofactor artificial metalloenzymes. The metal sites each largely retain their original structure and activity, as revealed through spectroscopy and electrochemical analyses. However, there are some changes observed at each center within the multimetallic constructs, indicating a minimal degree of perturbation induced upon introducing the second cofactor. Specifically, attachment of Co^MBQ^ to the M^Rd^ scaffold changed the pH dependence of the Co^MBQ^ onset potential (Fig. 5, inset), independent of whether M = Ni or Zn. Since this change occurs for both catalytically active and catalytically inert Rd species, it cannot derive from competing Ni^Rd^ activity, and instead suggests that proximity to the protein modifies the proton transfer to the cofactor or alters the local dielectric by changing hydrophobicity. On the other hand, the installation of Cu^MBQ^ to Ni^Rd^ increases the catalytic overpotential slightly. Though Cu^MBQ^ is catalytically inactive, the presence of an additional large hydrophobic group near the Ni^Rd^ center appears to increase the energy required to initiate proton reduction. We hypothesize that this may be due to steric bulk obstructing proton transfer to the active-site thiolate residues [26], restricting secondary sphere dynamics. The flexibility of the outer coordination sphere, as revealed through molecular dynamics simulations, shows some correlation with catalytic activity, as the hydrophobic valine residues flanking the Ni^Rd^ active site largely prevent direct solvent access [26, 27]. Changes in the local dielectric may also contribute. Ongoing work is aimed at understanding the molecular origin behind the minor perturbations observed in the Cu^MBQ^-bound Ni^Rd^. Nonetheless, the subtle shifts in activity and onset potential support the potential for independent tuning of each catalytic site and further development of this scaffold for cooperative catalysis.

### Construction of this highly tunable platform provides a new avenue to model hydrogenase

The M^MBQ^–M_o_–LM^Rd^ system described herein reveals important considerations for next-generation model design that recapitulate key elements of the native [NiFe] hydrogenase enzyme, which features three iron–sulfur clusters for electron transfer to the catalytic center. Divergent structures and environments around these clusters across [NiFe] hydrogenase variants have been proposed to impact the O_2_-tolerance, activity, and catalytic bias of the enzyme [48–50] without necessitating a significant change in the structure of the active site [51]. For example, in the newly characterized Huc hydrogenase from *Mycobacterium smegmatis*, which carries out hydrogen oxidation from ambient air, all clusters have a [3Fe–4S] configuration with high potentials that likely play a role in tuning the overpotential and enabling endogenous activity [52]. The O_2_-tolerant [NiFe] hydrogenases contain a unique proximal [4Fe-3S] cluster ligated by six cysteine residues instead of four [53, 54], allowing the cluster to donate an extra electron for O_2_ reduction [55]. Mutagenesis studies have underpinned the importance of this modified cluster for activity under prolonged O_2_ exposure [56, 57]. The medial and distal clusters also have been shown to play a role in modifying activity. For example, conversion of the high potential, medial [3Fe–4S] cluster to a [4Fe–4S] cluster in *Desulfovibrio fructosovorans* [NiFe] hydrogenase decreased the reduction potential by approximately 300 mV and caused a 38% reduction in H_2_ oxidation activity and a 60% increase in H_2_ evolution activity [58]. Likewise, activity differences are seen when the distal cluster ligation or secondary coordination sphere is varied [16, 59, 60]. However, while these isolated examples highlight the importance of the auxiliary clusters, we lack a predictive understanding of the impact that the electron transport chain has on activity and the extent to which it impacts catalytic bias.

The M^MBQ^–Cu–LNi^Rd^ system offers an opportunity to investigate, in a rational manner, the role of an exogenous redox partner on catalytic activity. While synthetic iron–sulfur clusters often suffer from degradation in protic solutions and irreversible redox transitions [61, 62], the MBQ compound has a reversible one-electron couple, is stable in aqueous solutions, and has a variable reduction potential depending on the identity of the central metal [31, 32]; as such, we suggest it may serve as a functional, if not structural, mimic of the iron–sulfur clusters seen in hydrogenases. While the presence of Cu^MBQ^ only minimally perturbed the activity of Ni^Rd^, likely due to the mismatch between the Cu(II/I) couple and the Ni^Rd^ onset of catalysis, and the Co^MBQ^ was independently active for H_2_ evolution, a benefit of the M^MBQ^–Cu–LNi^Rd^ platform lies in its modularity and tunability. The metal centers of MBQ, the MBQ itself, the linker, and the Rd protein can all be independently varied, with the potential for systematic investigations of electronic interactions between the added metallocofactor and the catalytic center of Ni^Rd^. Moreover, direct linkage to the protein via the bimetallic site will be pursued, leveraging our observation of minor amounts of MBQ binding to Cys31. These future studies will help elucidate the role of electron relays on catalysis in natural systems.

## Conclusions

In this work, multicofactor artificial metalloenzyme constructs have been developed by incorporating synthetic complexes based on a macrocyclic biquinazoline ligand near the active site of Ni^Rd^ (M^MBQ^–Cu–LNi^Rd^), with the goal of modeling bifunctional enzymatic systems found in nature. The introduction of M^MBQ^ into M^Rd^ did not alter the structure of the Ni^Rd^ active site or M^MBQ^. Both Co^MBQ^ and the Ni^Rd^ active site retained activity for hydrogen evolution in the Co^MBQ^–Cu–LNi^Rd^ construct, while control samples using Cu^MBQ^ or Zn^Rd^ were used to localize activity. Only minor changes in activity and onset potential were observed relative to the isolated components, indicating the presence of two independent active sites on a single molecular scaffold. This new platform offers the potential for development of robust, artificial metalloenzymes capable of tandem or coupled catalysis along with a robust and customizable system to help understand the roles of electron transport chains in native enzymes.

## Supplementary Information

Below is the link to the electronic supplementary material.

Supplementary file1 (PDF 3297 KB)

## Data Availability

Availability of data and materials Data are provided within the manuscript or supplementary information files. Raw data sets are available upon request. The X-ray datasets is available at the Edmond data repository: https://edmond.mpg.de/privateurl.xhtml?token=78844201-c6ee-4973-946b-2101239d5b03.

## Abbreviations

M^Rd^: Metal-substituted rubredoxin, where M = Fe, Ni, Zn
M^MBQ^: Macrocyclic biquinazoline ligand
CODH(s): Carbon monoxide dehydrogenases
ACS: Acetyl CoA synthase
ATP: Adenosine triphosphate
DCM: Dichloromethane
