# Supplementary material for: A semisynthetic, multicofactor artificial metalloenzyme retains independent site activity
Source: J Biol Inorg Chem. 2025 Feb 1;30(1):13–23. doi: 10.1007/s00775-025-02095-z (PMC11914312; doi:10.1007/s00775-025-02095-z)
Supplement: Supplementary file 1 — Supplementary file1 (PDF 3297 KB) [file 775_2025_2095_MOESM1_ESM.pdf]

A semisynthetic, multicofactor artificial metalloenzyme retains independent site activity

Ashlee E. Wertz[1], Ilmari Rosenkampff[2], Philippe Ibouanga[3,4], Matthias Huber[3,5], Corinna R. Hess*[3,5], Olaf Rüdiger*[2], Hannah S. Shafaat*[1,6]

[1]*Department of Chemistry and Biochemistry, The Ohio State University 100 W 18th Ave, Columbus, OH 43210*

[2]*Max Planck Institute for Chemical Energy Conversion, Stiftstrasse 34–36, D-45470 Mülheim an der Ruhr, Germany*

[3]*Technical University of Munich, Department of Chemistry and Catalysis Research Center, 85748 Garching, Germany*

[4]*Institut de Chimie Moléculaire et des Matériaux d'Orsay, Université Paris-Saclay, CNRS, 91405 Orsay, France*

[5]*Faculty of Chemistry and Pharmacy, University of Regensburg, 93053 Regensburg, Germany*

[6]*Department of Chemistry and Biochemistry, University of California, Los Angeles 607 Charles E. Young Drive East, Los Angeles, CA 90095*

Supplemental Information

Characterization of linker (**L**)………………………………………………………..………..……..**S3**

Supplemental Figures………………………………………………………..………………**S4-S22**

S1: ^{1}H NMR of the linker in $CDCl_3$
S2: ^{13}C NMR of the linker in $CDCl_3$
S3: Linker ESI-MS
S4: UV-Vis of linker attachment to Ni^{Rd}
S5: M^{MBQ}-Cu-LM^{Rd} formation as monitored by UV-Vis spectroscopy
S6: XPS of Co^{MBQ} covalently attached on a glassy carbon plate
S7: Three types of modified electrodes used in the electrochemical studies
S8: Complex formation control CVs
S9: Ni K-edge XANES of Cu^{MBQ}-Cu-LNi^{Rd} and Ni^{Rd}
S10: CVs of LCu, L-Co^{MBQ}, and LCu-Co^{MBQ}
S11: First derivatives of the Co^{MBQ}-Cu-LM^{Rd} CVs at pH 4.5
S12. pH dependence of Co^{MBQ} constructs
S13: Co^{MBQ}-Cu-LNi^{Rd} stability
S14: UV-vis of Co^{MBQ} and Cu^{MBQ} from pH 3-10
S15: CVs with homogenous Ni^{Rd}, Co^{MBQ}-Cu-LZn^{Rd}, and Co^{MBQ}-Cu-LNi^{Rd} solutions
S16: Linear parts of the adsorption time traces for Ni^{Rd}, Co^{MBQ}-Cu-LZn^{Rd}, and Co^{MBQ}-Cu-LNi^{Rd} samples
S17: CVs of L-Cu^{MBQ}, LCu-Cu^{MBQ}, and a blank PG electrode
S18: pH dependence of Cu^{MBQ}-Cu-LM^{Rd} constructs
S19: Cu^{MBQ}-Cu-LNi^{Rd} stability

Supplemental Tables………………………………………………………..……………**S23-S24**

S1: Inductively-coupled plasma mass spectrometry results
S2: Parameters from the EXAFS fits of Co, Ni, and Zn K-edge EXAFS

Supplemental Scheme……………………………………………………..………………**S25**

S1: Synthesis of thioether linker

Appendix 1………………………………………………………………………..…..………**S26**

Supplemental References………………………………………….…………………………**S27**

Characterization of linker (L)

All NMR spectra were measured at room temperature using a Bruker Avance Ultrashield 400 (400 MHz for ^{1}H, 101 MHz for ^{13}C) spectrometer. All chemical shifts are reported in δ-scale as parts per million [ppm] (multiplicity, coupling constant J, number of protons) relative to the solvent ($CDCl_3$) residual peaks as the internal standard. Coupling constants J are given in Hertz [Hz]. Abbreviations used for signal multiplicity: ^{1}H NMR: s = singlet, t = triplet, p = pentet, dt = doublet of triplets, and m = multiplet. Electrospray ionization mass spectra (ESI-MS) were obtained on a Bruker MicroTOF-Q instrument.

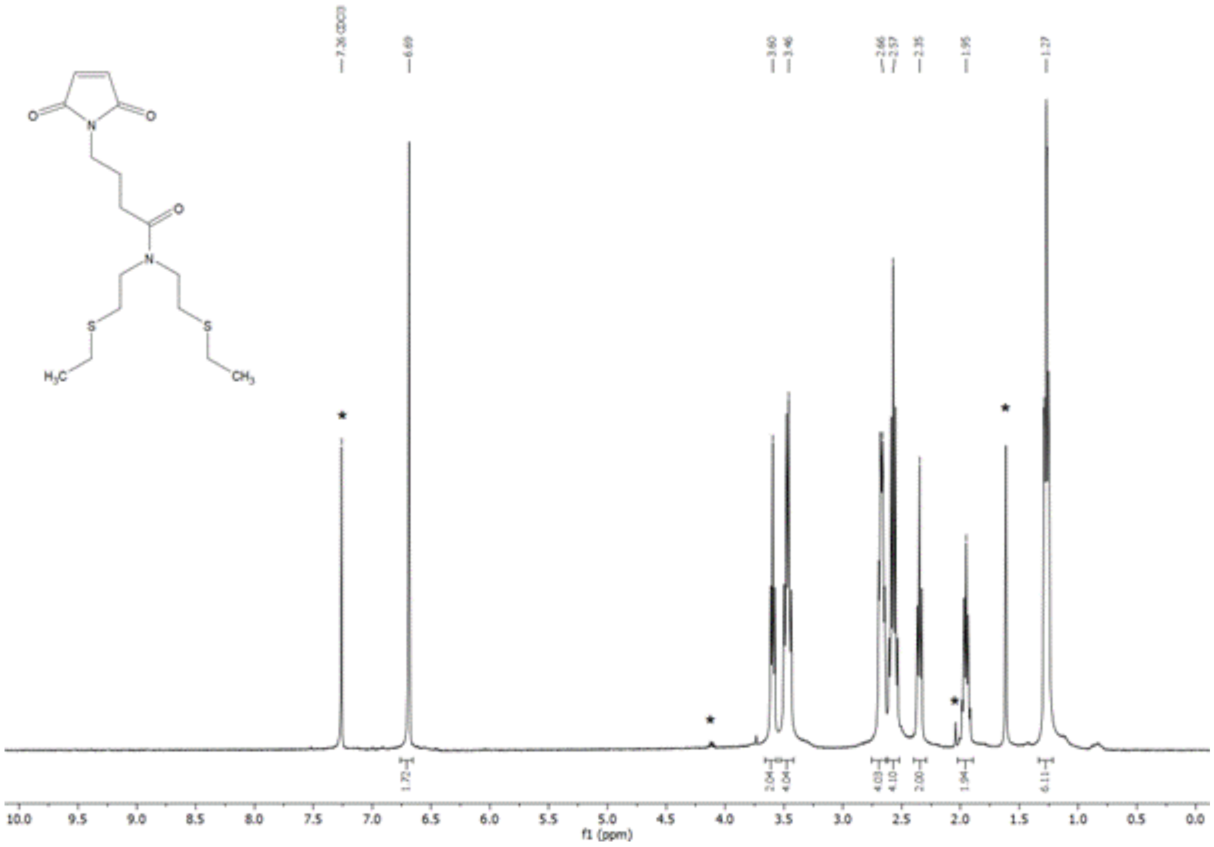

Figure S1. ^{1}H NMR of the linker, **L**, in $CDCl_3$. $CDCl_3$, residual water, and EtOAc signals are marked with asterisks.

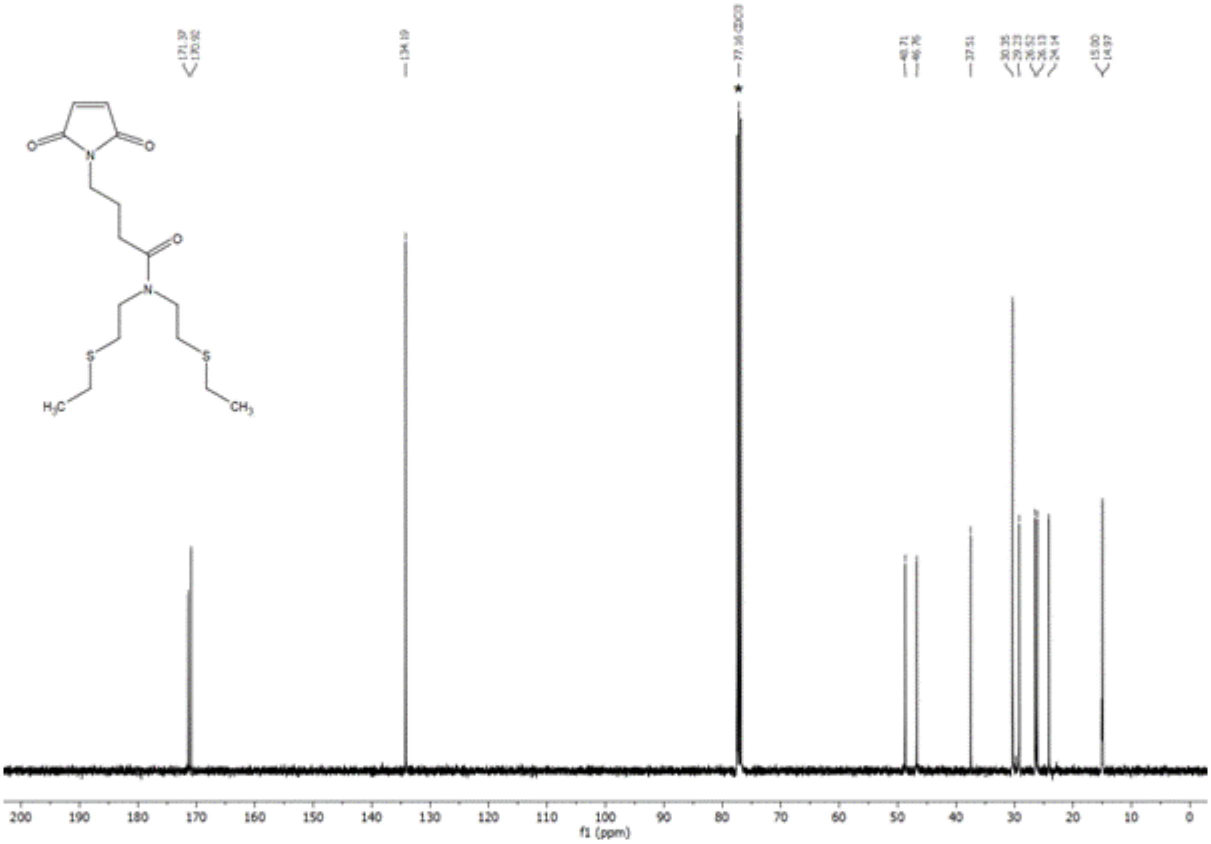

Figure S2. ^{13}C NMR of the linker, **L**, in $CDCl_3$. $CDCl_3$ signal marked with an asterisk.

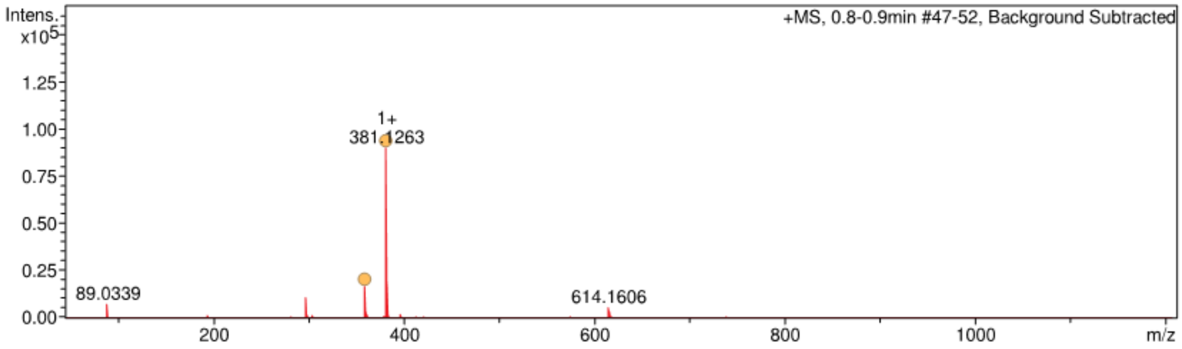

Meas. m/z	#	Ion Formula	m/z	err [mDa]	err [ppm]	mSigma
359.144228	1	C16H27N2O3S2	359.145761	1.5	4.3	7.0
381.126273	1	C16H26N2NaO3S2	381.127705	1.4	3.8	2.0

Figure S3. ESI-MS of **L**

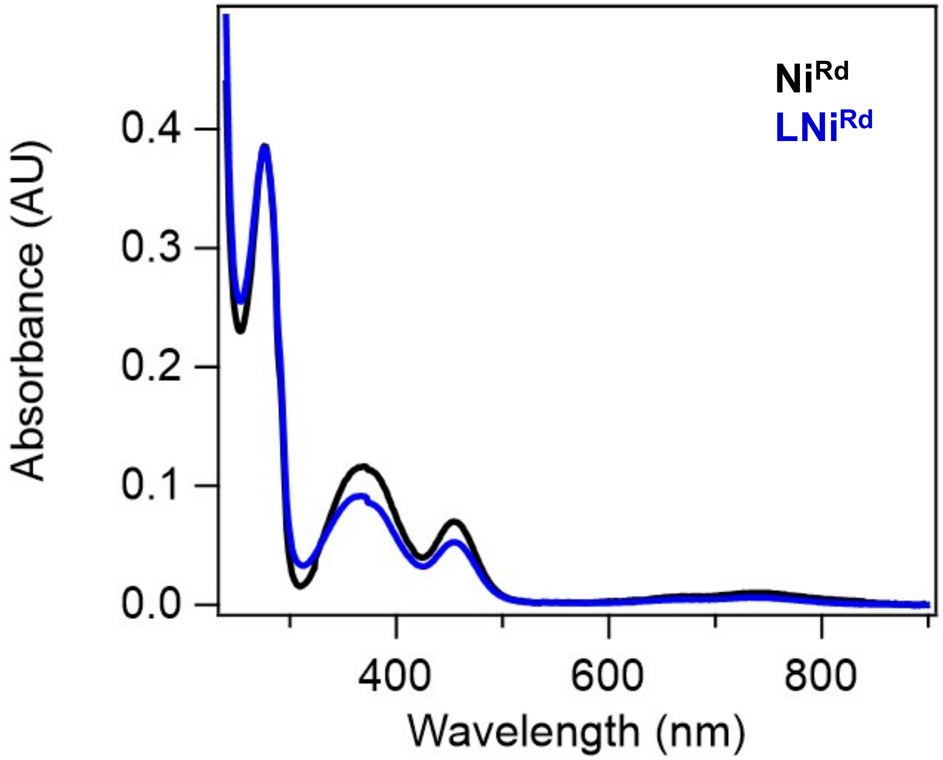

Figure S4. Optical spectra showing linker attachment to NiRd. Samples measured in 50 mM Tris buffer, pH 8.0.

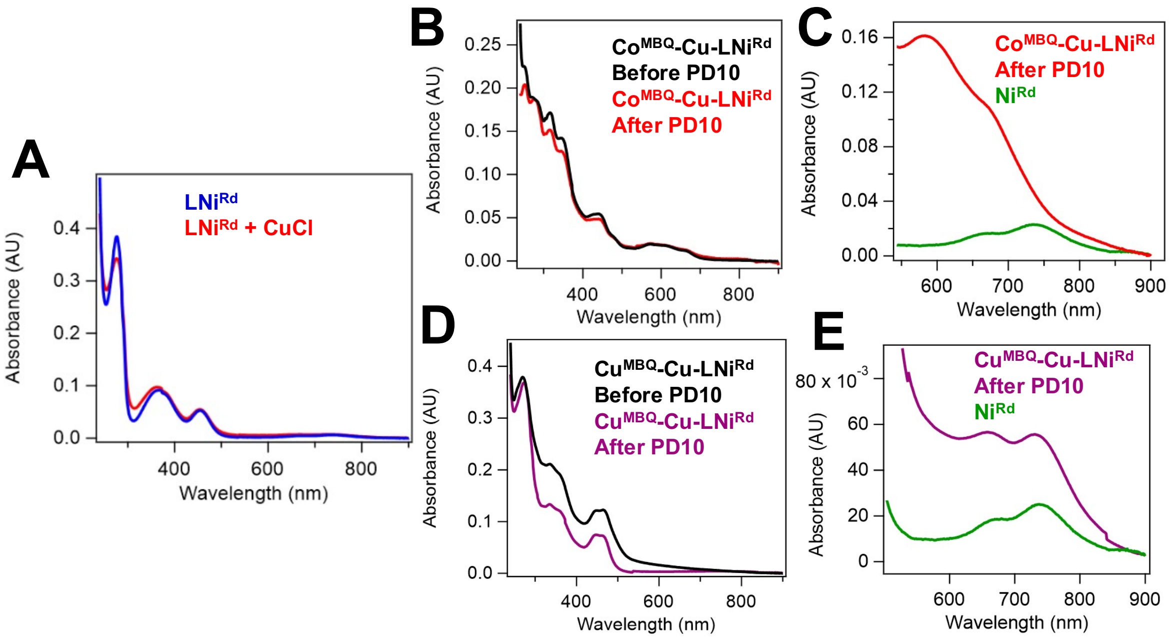

Figure S5. Optical spectra showing M^{MBQ}-Cu-LMRd formation. All spectra are shown in 50 mM Tris buffer, pH 8.0. (**A**) UV-Vis spectra of LNiRd and LNiRd with 2-fold excess CuCl added. (**B**) Co^{MBQ}-Cu-LNiRd construct formation before and after a PD10 desalting column. (**C**) Zoomed-in view of spectral region of d-d absorption features for NiRd and Co^{MBQ}-Cu-LNiRd. (**D**) Cu^{MBQ}-Cu-LNiRd construct formation before and after a PD10 desalting column. (**E**) Zoomed-in view of spectral region of d-d absorption features for NiRd and Co^{MBQ}-Cu-LNiRd. The concentration of NiRd present was determined using ε_{740} = 390 $M^{-1}cm^{-1}$ for NiRd. The concentration of M^{MBQ} was determined by using Co^{MBQ}ε_{455} = 8550 $M^{-1}cm^{-1}$ or Cu^{MBQ}ε_{455} = 15700 $M^{-1}cm^{-1}$ and subtracting the absorbance present from NiRd (ε_{455} = 3400 $M^{-1}cm^{-1}$)

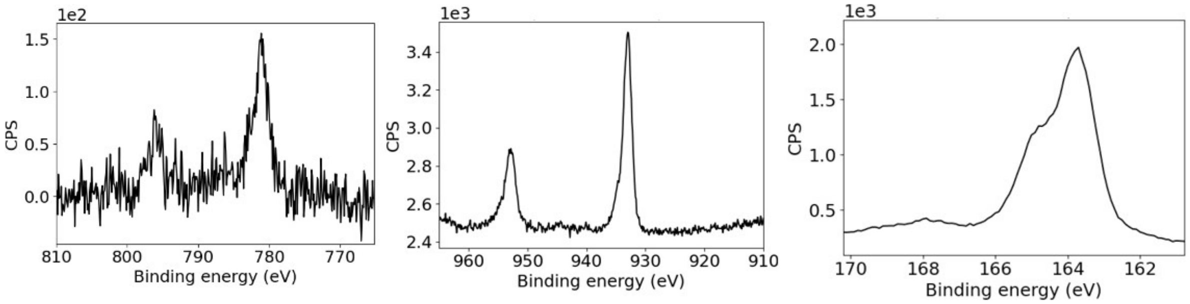

Figure S6. XPS of LCu-Co^{MBQ} covalently attached on a glassy carbon plate, showing transitions from Co (l*eft*), Cu (*middle*), and S (*right*).

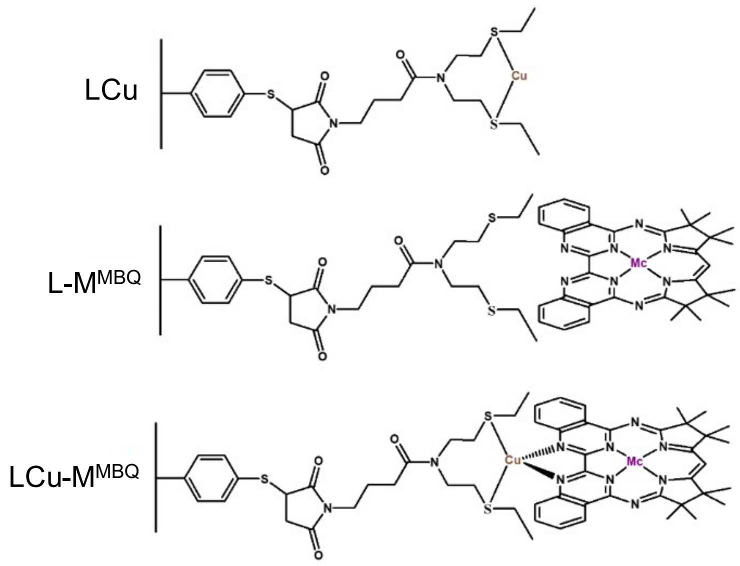

Figure S7. Three types of modified electrodes used in the study: electrode LCu, containing the linker and Cu^{I}; electrode L-M^{MBQ}, containing the linker, and M^{II}Mabiq; and electrode LCu-M^{MBQ}, containing the linker, Cu^{I}, as well as M^{II}Mabiq.

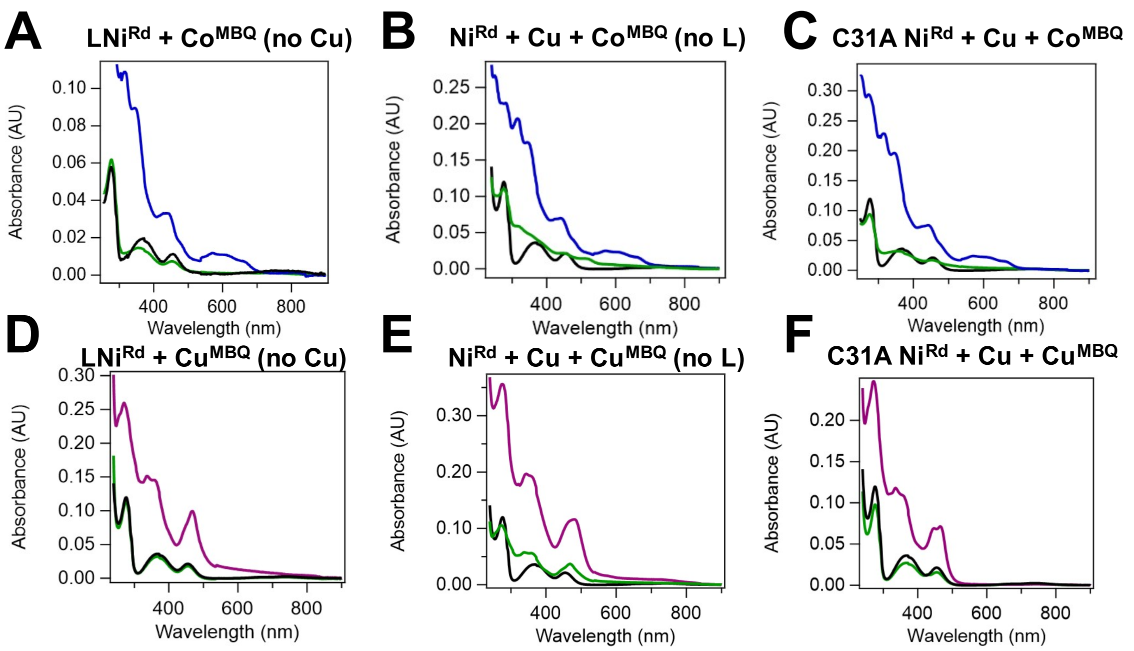

Figure S8. Optical spectra to test for product formation. Control samples prior to the desalting column for Co^{MBQ} (*blue*), Cu^{MBQ} (*purple*), LNi^{Rd} (*black*), and compounds after the PD-10 desalting column (*green*). All spectra were obtained in 50 mM Tris buffer, pH 8.0. (**A**) LNi^{Rd} and 5x Co^{MBQ} with no CuCl added. (**B**) Ni^{Rd} + 2x CuCl + 5x Co^{MBQ} with no linker covalently attached to the protein. (**C**) C31A Ni^{Rd} + 2x CuCl + 5x Co^{MBQ} (**D**) LNi^{Rd} and 5x Cu^{MBQ} with no CuCl added. (**E**) Ni^{Rd} + 2x CuCl + 5x Cu^{MBQ} with no linker covalently attached to the protein. (**F**) C31A Ni^{Rd} + 2x CuCl + 5x Cu^{MBQ}.

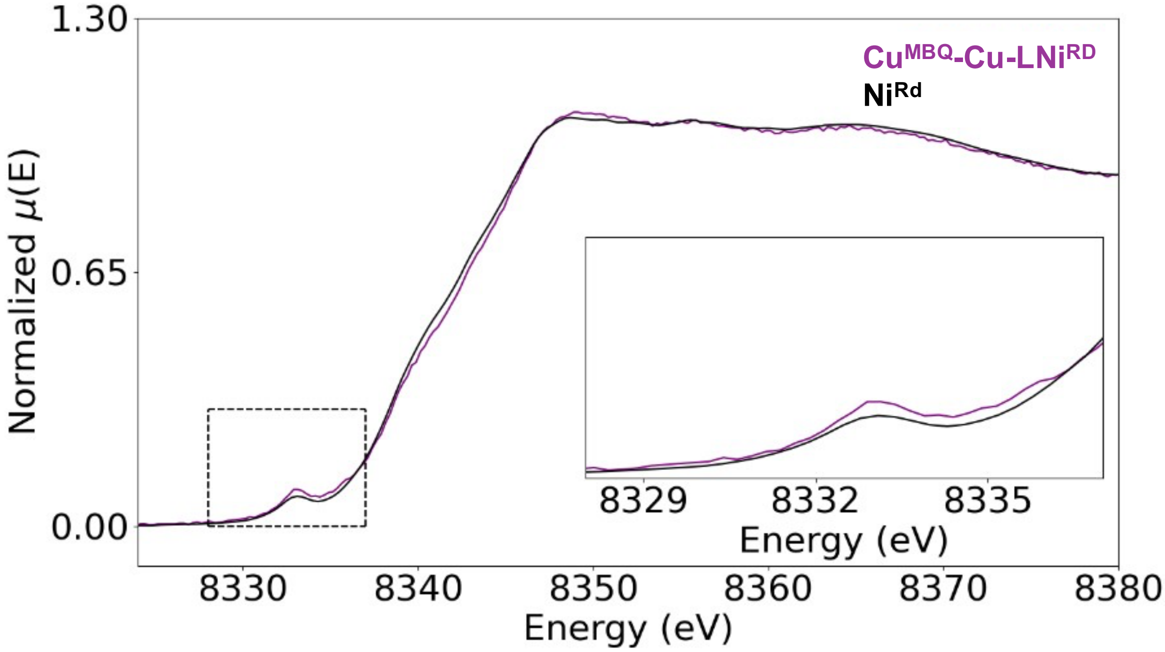

Figure S9. Ni K-edge XANES of Cu^{MBQ}-Cu-LNi^{Rd} and Ni^{Rd}.

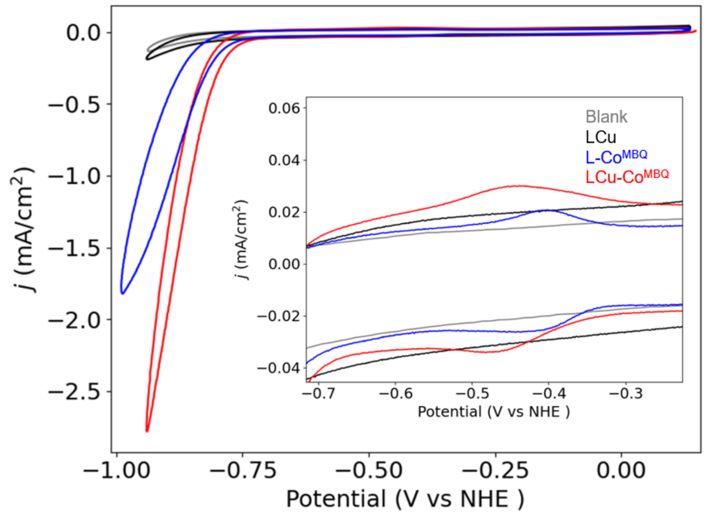

Figure S10. Cyclic voltammograms of LCu, L-Co^{MBQ}, and LCu-Co^{MBQ} in 25 mM CHaMp buffer, pH 4.5 (ν = 100 mV/s).

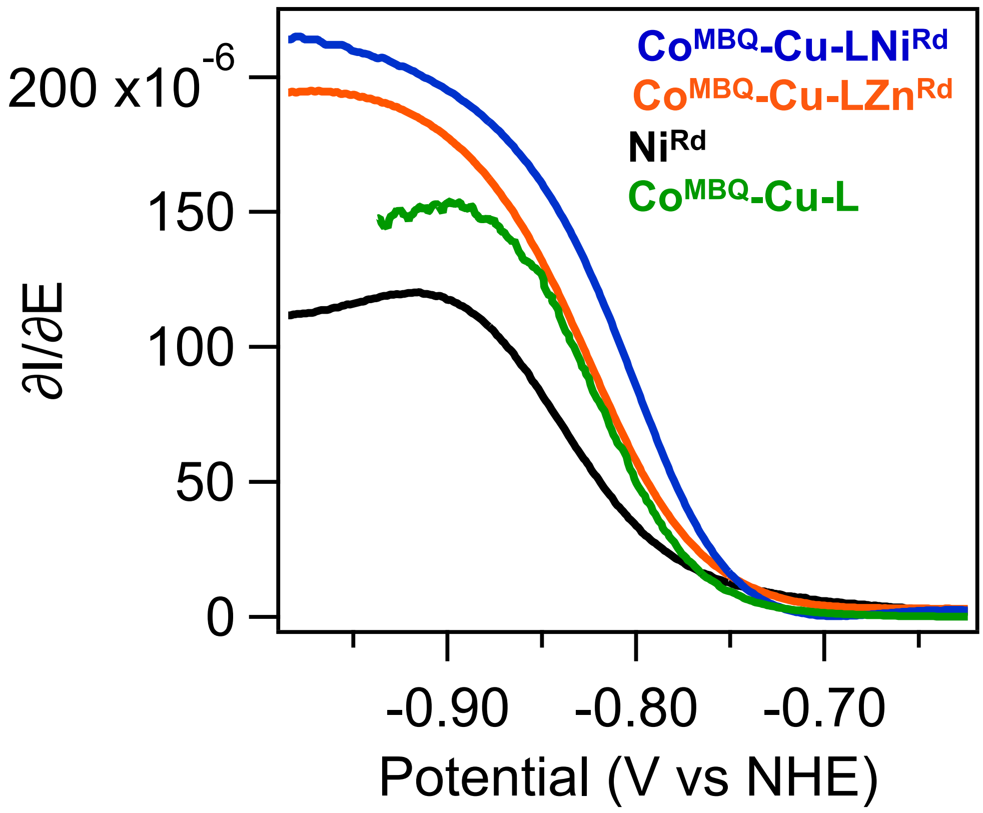

Figure S11. First derivatives of the cyclic voltammograms of the Co^{MBQ}-Cu-M^{Rd} samples (as indicated) in 25 mM CHaMp buffer, pH 4.5.

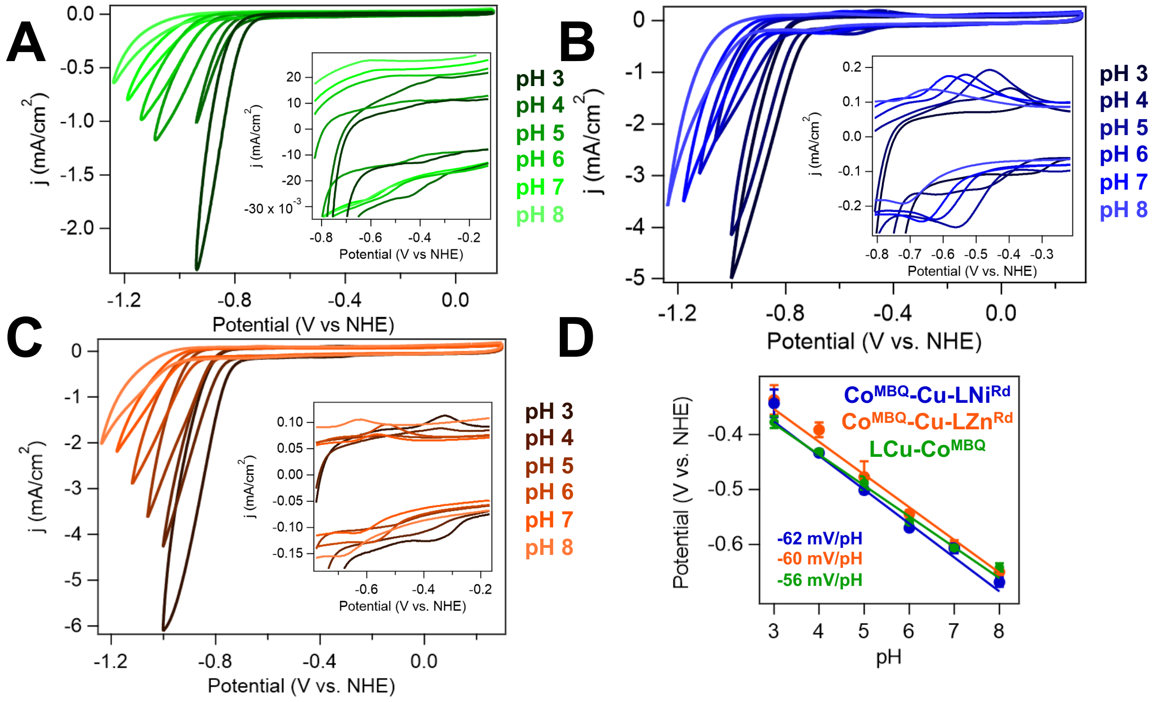

Figure S12. pH-dependent CVs of the Co^{MBQ} constructs: (**A**) LCu-Co^{MBQ}; (**B**) Co^{MBQ}-Cu-LNiRd construct; and (**C**) Co^{MBQ}-Cu-LZnRd construct. CVs were run in 25 mM CHaMp buffer at the indicated pH (ν = 100 mV/s). (**D**) Co^{MBQ} midpoint potential as a function of pH.

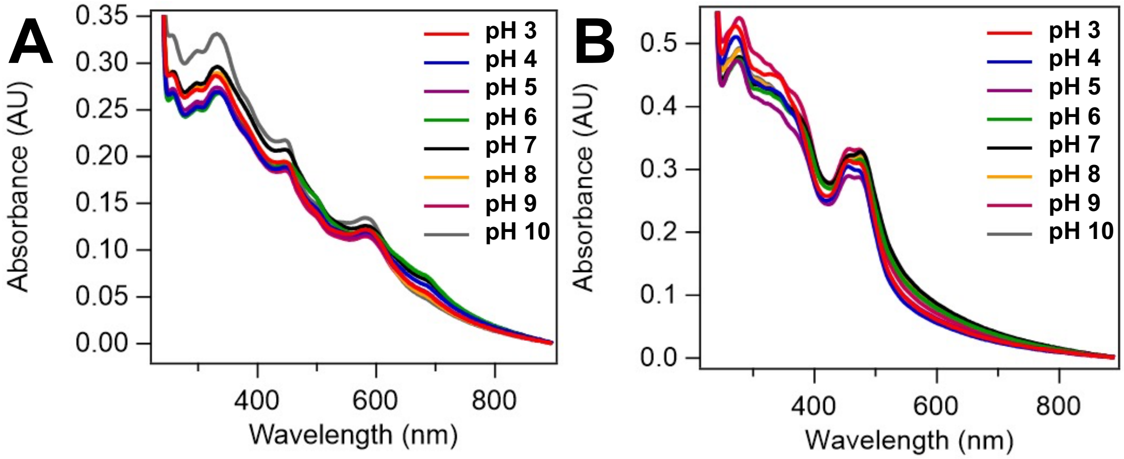

Figure S14. Optical spectra as a function of pH of (**A**) Co^{MBQ} and (**B**) Cu^{MBQ} from pH 3-10 in 25 mM CHaMp buffer.

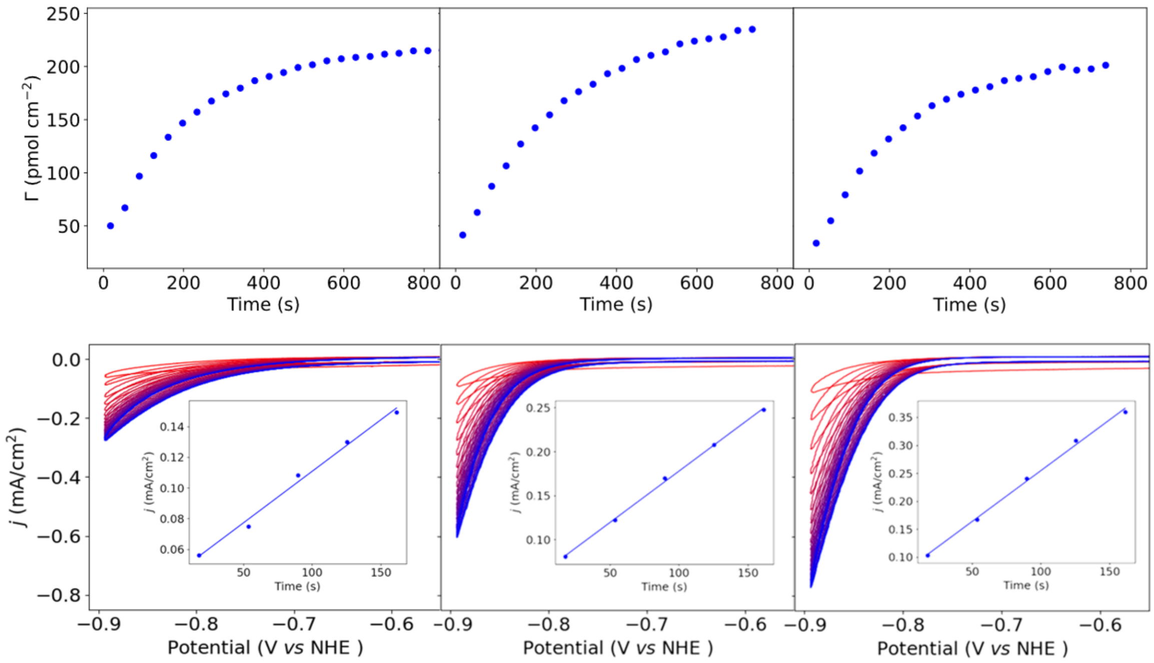

Figure S15. CVs with homogenous Ni^{Rd} (*bottom left*), Co^{MBQ}-Cu-LZn^{Rd} (*bottom middle*), and Co^{MBQ}-Cu-LNi^{Rd} (*bottom right*) solutions measured with a graphitic RDE with a rotation rate of 1000 rpm together with simulated coverages obtained with Equation S1 (*top graphs*). Current densities of the cathodic scans at -0.88 V vs NHE during the first few cycles are shown in the insets, with linear fits for TOF analysis. Experiments start from red and proceed to the blue scans. Experiments were run in 25 mM CHaMp buffer, pH 3.0 (ν = 100 mV/s).

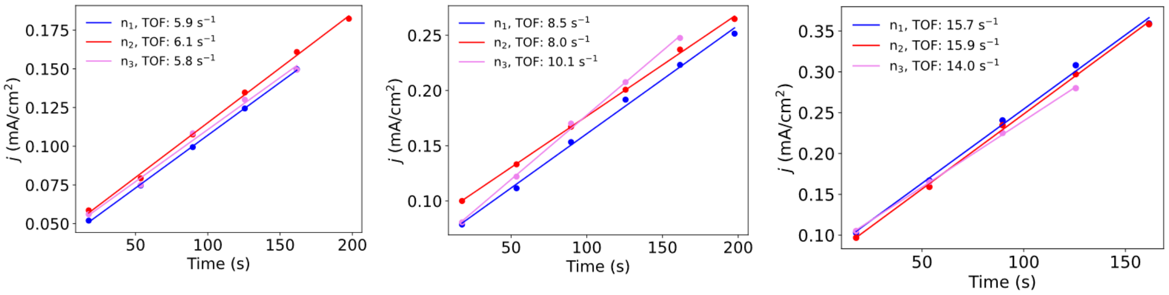

Figure S16. Linear parts of the adsorption time traces for Ni^{Rd} (*left*), Co^{MBQ}-Cu-LZn^{Rd} (*middle*), and Co^{MBQ}-Cu-LNi^{Rd} (*right*) samples with a rotation rate of 1000 rpm at -0.88 V *vs* NHE. pH: 3. TOF(Ni^{Rd}) = 5.9 ± 0.1 s^{-1}, TOF(Co^{MBQ}-Cu-LZn^{Rd}) = 8.9 ± 0.9 s^{-1}, TOF(Co^{MBQ}-Cu-LNi^{Rd}) = 15.2 ± 0.9 s^{-1}.

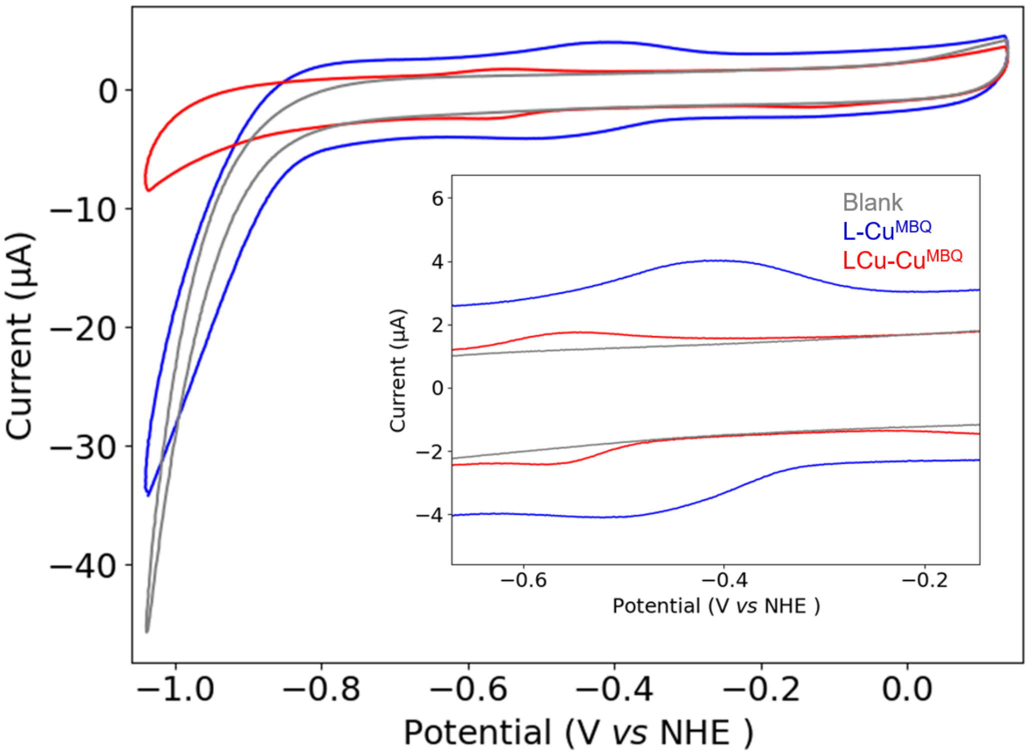

Figure S17. Cyclic voltammograms of L-Cu^{MBQ}, and LCu-Cu^{MBQ} in 25 mM CHaMp buffer, pH 4.5 (ν = 100 mV/s). Unlike L-Cu^{MBQ}, the electrode LCu-Cu^{MBQ} was rinsed with DMF after the attachment, removing nonspecifically-bound Cu^{MBQ} from the electrode. This explains the larger capacitive current and reversible wave in L-Cu^{MBQ} compared to LCu-Cu^{MBQ}.

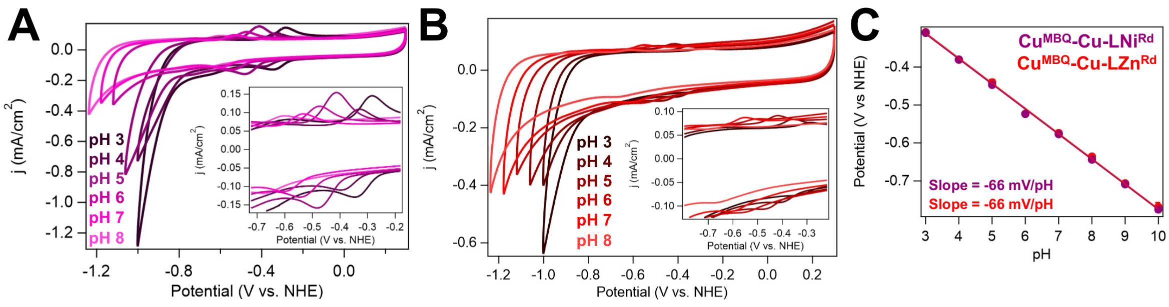

Figure S18. pH-dependent CVs of the Cu^{MBQ} constructs: (**A**) Cu^{MBQ}-Cu-LNiRd construct; and (**B**) Cu^{MBQ}-Cu-LZnRd construct. CVs were run in 25 mM CHaMp buffer at the indicated pH (ν = 100 mV/s). (**C**) Cu^{MBQ} midpoint potential as a function of pH.

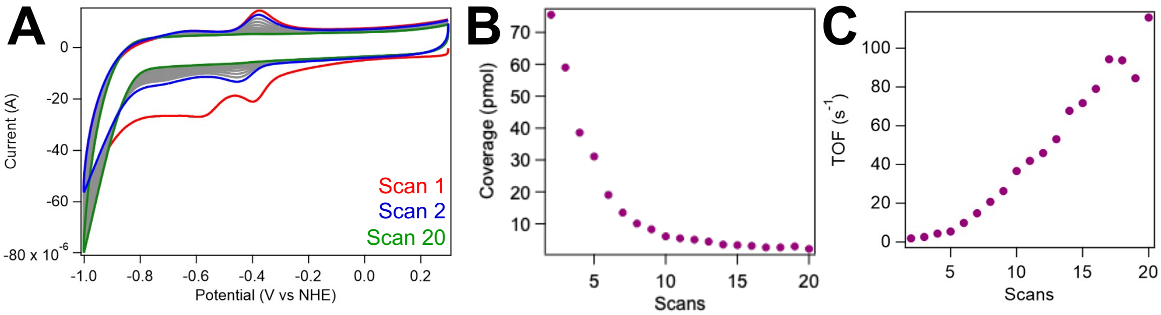

Figure S19. Cu^{MBQ}-Cu-LNi^{Rd} stability. (**A**) CVs of Cu^{MBQ}-Cu-LNi^{Rd} over 20 scans in 25 mM CHaMp buffer, pH 4.5 (ν = 100 mV/s). (**B**) Coverage and (**C**) TOFs using the qPFE method extracted from the 20 scans shown in (**A**).

Construct	ppm Zn	ppm Cu	ppm Co	ppm Ni
**Co^{MBQ}-Cu-LNi^{Rd}**	0	140.3	126.4	131.1
**Co^{MBQ}-Cu-LZn^{Rd}**	153.8	97.2	91.1	0
**Cu^{MBQ}-Cu-LNi^{Rd}**	0	39.4	0	57.3
**Cu^{MBQ}-Cu-LZn^{Rd}**	13.1	29.2	0	0

Table S1. Inductively coupled plasma mass spectrometry results.

Co^{MBQ}-Cu-LNi^{Rd}, Co-EXAFS	N	σ^2[Å^2]	R_{input}[Å]	$R_{calculated}$[Å]	ΔR[Å]
Co-N_1	4	0.0014 ± 0.0004	1.91	1.89	-0.02 ± 0.01
Co-O_1	1	0.007 ± 0.006	2.24	2.25	0.01± 0.04
Co-C_2	8	0.005 ± 0.001	2.89	2.86	-0.03 ± 0.02
Co-N_1-C_2	8	0.0021 ± 0.0006	3.09	2.97	-0.12 ± 0.07
Co-N_3	4	0.0021 ± 0.0006	3.30	3.24	-0.06 ± 0.02
Co^{MBQ}-Cu-LZn^{Rd}, Co-EXAFS					
Co-N_1	4	0.0012 ± 0.0005	1.91	1.89	-0.02 ± 0.01
Co-O_1	1	0.002 ± 0.004	2.24	2.26	0.02 ± 0.03
Co-C_2	8	0.007 ± 0.003	2.89	2.88	-0.01 ± 0.03
Co-N_1-C_2	8	0.002 ± 0.001	3.1	3.2	0.1 ± 0.2
Co-N_3	4	0.002 ± 0.004	3.30	3.19	-0.11 ± 0.03
Co^{MBQ}-Cu-LNi^{Rd}, Ni-EXAFS					
Ni-S_1	4	0.0025 ± 0.001	2.28	2.26	-0.02 ± 0.01
Cu^{MBQ}-Cu-LNi^{Rd}, Ni-EXAFS					
Ni-S_1	4	0.0028 ± 0.0007	2.38	2.38	0.00 ± 0.01
Co^{MBQ}-Cu-LZn^{Rd}, Zn-EXAFS					
Zn-S_1	4	0.0025 ± 0.0004	2.340	2.340	0.000 ± 0.007
Cu^{MBQ}-Cu-LZn^{Rd}, Zn-EXAFS					
Zn-S_1	4	0.0028 ± 0.0002	2.340	2.342	0.002 ± 0.003

Table S2. Parameters from the EXAFS fits of Co, Ni, and Zn K-edge EXAFS of each of the M^{MBQ}-Cu-LM^{Rd} constructs. The amplitude reduction factor was set to 0.9. Calculated shifts to experimental edges were between -1.3 and 5.2 eV and the R-factors between 0.003 and 0.017. The guessed path-distances ($R_{crystal}$) for Co EXAFS are evaluated from the crystal structure of [Co(Mabiq)(THF)]PF_6, for Ni EXAFS from the crystal structure of the *Desulfovibrio desulfuricans* Ni^{Rd} (PDB accession code: 6NW0), and for Zn EXAFS from the crystal structure of a zinc finger protein (PDB accession code: 4QF3).[1-3]

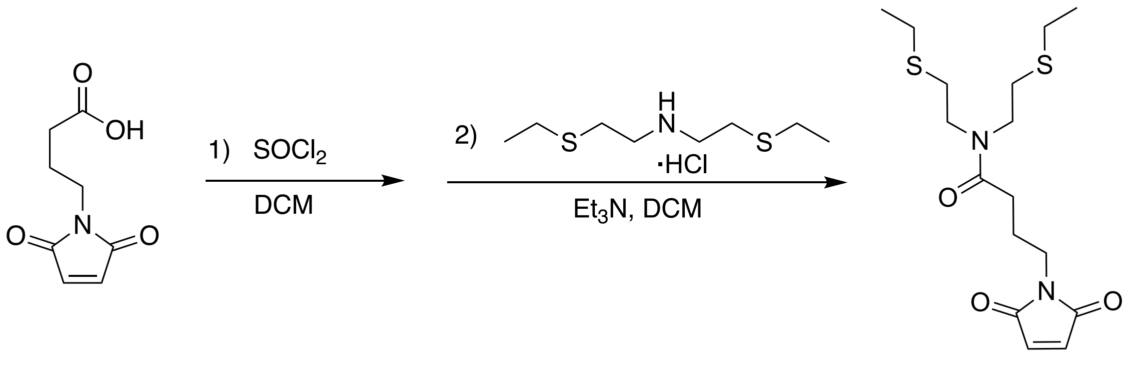

L

Scheme S1. Synthesis of thioether linker, **L.**

Appendix 1

TOF measurements of homogenous samples

Turnover frequencies of the protein constructs were obtained as reported in ref. 4. For clarity, a summary of the method is provided here. The turnover frequency (TOF) of an enzyme in aqueous solution can be measured by utilizing mass transport of the enzyme towards an initially bare electrode. In this experiment, the working electrode is rotated, which enables correlating an increase in current density with well-known equations of convective-diffusion governed mass transport. The starting point is the expression for the catalytic current density,

$$j(t) = 2 \cdot \mathrm{F} \cdot TOF \cdot \Gamma, \quad \text{(S1)}$$

where TOF is the potential-dependent turnover frequency (s^{-1}), F is the Faraday constant (C mol^{-1}) and Γ is the enzyme coverage (mol m^{-2}) on the electrode. In time, the catalytic current changes with increased enzyme coverage:

$$\frac{dj(t)}{dt} = 2 \cdot \mathrm{F} \cdot \mathrm{TOF} \cdot \frac{d\Gamma(t)}{dt}. \quad \text{(S2)}$$

Observing that $d\Gamma(\mathrm{t})/\mathrm{dt}$ is flux (mol s^{-1} m^{-2}) of the enzyme that arises from the concentration gradient between the bulk solution and the vicinity of the bare electrode surface and from the rotation of the electrode, it can be expressed as

$$\frac{d\Gamma}{dt} = m \cdot c_{\mathrm{bulk}}, \quad \text{(S3)}$$

where c_{bulk} is the bulk enzyme concentration (M) and *m* is the mass-transport coefficient (m s^{-1}),

$$m = 0.62 \cdot D_{enz}^{2/3}\, \omega^{1/2} \nu^{-1/6}. \quad \text{(S4)}$$

Here, D_{enz} is the diffusion coefficient of the enzyme (m^2 s^{-1}), ω is the rotation rate of the electrode (s^{-1}), and ν is the kinematic viscosity of water, taken as 0.8927 mm^2 s^{-1} at 25 °C. Equation S3 holds in the beginning of the experiment, when the electrode surface is still almost free of adsorbed enzyme. Consequently, the increase in the current density in the beginning of the experiment is linear in time.

The diffusion coefficient can be expressed with the Stokes-Einstein equation,

$$D_{enz} = \frac{k_B T}{6\pi\mu r}, \quad \text{(S5)}$$

where *r* is the hydrodynamic radius of the protein and μ is the dynamic viscosity of water, taken to be 0.89 mPa·s at 25 °C. The hydrodynamic radius of the M^{MBQ}-Cu-LM^{Rd} constructs was estimated to be 14.3 Å, which is the hydrodynamic radius of a Zn substituted Rd mutant, A51C, at 25 °C.[5] This gave a diffusion coefficient of 1.7×10^{-6} cm^2 s^{-1}.

Turnover frequency was obtained by rearranging Equation S2 with the help of Equation S3 into

$$\mathrm{TOF} = \frac{dj/dt}{2 \cdot \mathrm{F} \cdot m \cdot c_{\mathrm{bulk}}}. \quad \text{(S6)}$$

The initial increase of current density in time (d*j*/dt) was evaluated from a linear fit to the current densities measured at -0.88 V *vs*. NHE of the first few cathodic scans (Figure S15). Potential was cycled during the experiments with a scan rate of 100 mV/s, while

rotating the electrode at 1000 rpm. In the derivation of the Levich flux equation, Equation S3, the flux is expressed with respect to the geometrical surface area, or the area perpendicular to the electrode.[6] For this reason, experimental current densities have been obtained by dividing the current with the geometrical surface area of the electrode.

After calculation of the *TOF*, the coverage of the homogenous protein samples was evaluated with the equation

$$\Gamma(t) = \frac{j(t)}{2 \cdot F \cdot TOF} \tag{S7}$$

by taking the current densities at -0.88 V *vs*. NHE from each measured cathodic scan during cyclic voltammetry (Figure S15).

Supplemental References

1. Kaspar, M., Altmann, P.J., Pöthig, A., Sproules, S. and Hess, C.R., **2017**. A macrocyclic ‘Co^0’ complex: the relevance of ligand non-innocence to reactivity. *Chemical Communications*, *53*(53), 7282-7285.
2. Slater, J.W., Marguet, S.C., Gray, M.E., Monaco, H.A., Sotomayor, M. and Shafaat, H.S., **2019**. Power of the secondary sphere: modulating hydrogenase activity in nickel-substituted rubredoxin. *ACS Catalysis*, 9(10), 8928-8942.
3. Tallant, C., Valentini, E., Fedorov, O., Overvoorde, L., Ferguson, F.M., Filippakopoulos, P., Svergun, D.I., Knapp, S. and Ciulli, A., **2015**. Molecular basis of histone tail recognition by human TIP5 PHD finger and bromodomain of the chromatin remodeling complex NoRC. *Structure*, *23*(1), 80-92.
4. Santos, A., Duarte, A.G., Fedorov, A., Martinho, J.M. and Moura, I., **2010**. Rubredoxin mutant a51c unfolding dynamics: a Förster resonance energy transfer study. *Biophysical Chemistry*, *148*(1-3),131-137.
5. Bard, A.J., Faulkner, L.R. and White, H.S., **2001**. *Electrochemical methods: fundamentals and applications*, 2nd ed. John Wiley & Sons.
